# Early Local Activity in Temporal Areas Reflects Graded Content of Visual Perception

**DOI:** 10.3389/fpsyg.2016.00572

**Published:** 2016-04-25

**Authors:** Chiara F. Tagliabue, Chiara Mazzi, Chiara Bagattini, Silvia Savazzi

**Affiliations:** ^1^University of Verona and National Institute of Neuroscience, VeronaItaly; ^2^Perception and Awareness Laboratory, Department of Neurological, Biomedical and Movement Sciences, University of Verona, VeronaItaly; ^3^Cognitive Neuroscience Section, IRCCS Centro San Giovanni di Dio Fatebenefratelli, BresciaItaly

**Keywords:** access consciousness, EEG, ERPs, neural correlates, perceptual awareness scale, phenomenal consciousness, visual awareness

## Abstract

In visual cognitive neuroscience the debate on consciousness is focused on two major topics: the search for the neural correlates of the different properties of visual awareness and the controversy on the graded versus dichotomous nature of visual conscious experience. The aim of this study is to search for the possible neural correlates of different grades of visual awareness investigating the Event Related Potentials to reduced contrast visual stimuli whose perceptual clarity was rated on the four-point Perceptual Awareness Scale. Results revealed a left centro-parietal negative deflection (Visual Awareness Negativity; VAN) peaking at 280–320 ms from stimulus onset, related to the perceptual content of the stimulus, followed by a bilateral positive deflection (Late Positivity; LP) peaking at 510–550 ms over almost all electrodes, reflecting post-perceptual processes performed on such content. Interestingly, the amplitude of both deflections gradually increased as a function of visual awareness. Moreover, the intracranial generators of the phenomenal content (VAN) were found to be located in the left temporal lobe. The present data thus seem to suggest (1) that visual conscious experience is characterized by a gradual increase of perceived clarity at both behavioral and neural level and (2) that the actual content of perceptual experiences emerges from early local activation in temporal areas, without the need of later widespread frontal engagement.

## Introduction

Consciousness (or awareness) refers to the fact that, when we are awake, we have experiences. Since consciousness gained enough consideration to be investigated in the field of cognitive neuroscience, an intensive search for the neural correlates of consciousness (NCC) has been undertaken. The NCC has been defined by Koch (2004) as “the minimal set of neuronal events and mechanisms jointly sufficient for a specific conscious percept.”

Such NCC are usually investigated by contrasting neural responses to physically identical stimuli that are consciously perceived or not, the so-called contrastive analysis (Baars, 1988), used across different experimental paradigms in which visual awareness is manipulated (e.g., masking, change blindness, reduced-contrast stimuli, etc. For a review, see Koivisto and Revonsuo, 2010). fMRI studies have revealed that changes in conscious contents correlate with activation along the ventral visual pathway (e.g., Bar et al., 2001; Pins and Ffytche, 2003) with additional involvement of frontal and parietal areas (e.g., Lumer and Rees, 1999; Beck et al., 2001), revealing the key role of dorsal-ventral interactions for visual awareness. The temporal dynamics of such neural processing have been obtained by studying event-related brain potentials (ERPs), the electrical potential changes in response to a given sensory, motor or cognitive event (Luck, 2005). Recent ERP studies have found that conscious perception consistently correlates with an early component called Visual Awareness Negativity (VAN; Koivisto and Revonsuo, 2003), that is a negative amplitude difference wave between aware and unaware trials peaking at about 200 ms after stimulus onset in occipito-temporal sites (Koivisto et al., 2008), but also observed at central, fronto-polar (Wilenius-Emet et al., 2004) and occipital-parietal (Pitts et al., 2014) electrodes. The latency of this component is prolonged (up to 200 ms later) when the contrast of the stimuli is lowered (Ojanen et al., 2003). The VAN is usually followed by a later positive component, called Late Positivity (LP; Del Cul et al., 2007), another difference wave between aware and unaware conditions peaking between 300 and 400 ms after stimulus presentation in parietal and central sites. Finally, weaker evidence has been found also for an enhancement of P1 amplitude in response to aware trials at around 100–130 ms in the occipital sites (Pins and Ffytche, 2003), even if this early positive component might better reflect attention-related processes (Hillyard et al., 1998).

Importantly, it has been proposed (Block, 2005) that a distinction needs to be made between two components of consciousness: phenomenal consciousness, described as the “what-it-is-like” of the experience (the actual content), and access consciousness that is the ability to report, remember or act on such experiences. Accordingly, different NCC might reflect each one of these components (Block, 1996). Following the classification made by Block, Koivisto and Revonsuo (2010) have proposed that the two components typically found in ERP experiments to correlate with visual awareness (VAN and LP) may represent distinctive NCC of the different properties of consciousness. More specifically, given their latencies and topographies, the VAN, the earlier ERP component, has been interpreted (Koivisto and Revonsuo, 2010) as the neural correlate of phenomenal awareness, whereas the LP, the later ERP component, has been related to access awareness.

Together with the classification of consciousness in the phenomenal and the access components, another important issue that has to be taken into account when investigating visual awareness relates to the way the perceptual experience is reported. Studies on unconscious perception typically require the participants to report whether or not they saw a stimulus, thus measuring their conscious experiences in a dichotomous way (e.g., Baars, 1994). In this perspective, then, consciousness is considered as an all-or-none process. However, it has been argued that conscious perception is a complex phenomenon characterized by different degrees of clarity, thus needing more elaborated report measures to be adopted (Ramsøy and Overgaard, 2004).

In the light of these considerations, in order to obtain more detailed subjective reports Ramsøy and Overgaard (2004) developed a four-point scale to assess the clarity of perceptual experiences: the Perceptual Awareness Scale (PAS). The four points consistently used by the participants to judge their visual perceptions were: (1) no experience of the stimulus, (2) brief glimpse, (3) almost clear experience and (4) clear experience. The PAS proved to be the most exhaustive measure of visual awareness compared to other graded scales and showed a good correlation between performance and awareness, possibly implying that different cognitive processes actually take place for each level of the scale (Sandberg et al., 2010). The four categories of the PAS thus refer to the quality of the perceptual experience, differently from confidence ratings that mostly involve metacognitive knowledge about the perceptual content (see Sandberg et al., 2010 for a comparison of report methodologies).

Accordingly, an fMRI study (Christensen et al., 2006) investigated the neural correlates of the use of a three-point scale (clear, vague, no perceptual experience) to rate the clarity of visual experiences in response to briefly presented stimuli. Interestingly, the authors revealed that different levels of awareness correlated with different degrees but also with different patterns of brain activation. More specifically, reports of clear experiences activated a network including parietal, temporal, frontal, basal ganglia and thalamic areas, while reports of vague perception resulted in graded activation within the same network but also in specific activations in frontal and insular regions, not seen for reports of clear experiences. Also a recent MEG study (Andersen et al., 2015) found that, during a visual masking task, occipital sources in the VAN time range were more accurate in decoding visual awareness as assessed on the four categories of the PAS, providing further evidence that perceptual awareness may be best described as a graded phenomenon.

So far, just a few papers have used a graded scale to assess visual awareness using EEG. The most evident limitation is that not all the categories of the scale were actually investigated. For example, Melloni et al. (2011) studied how previous experience affected conscious perception of stimuli presented at different degradation levels. Participants had to rate target visibility on the PAS, but then the authors decided to recode the scale into a dichotomous scale and found that P200 amplitude was inversely modulated by perceptual awareness. More recently, in another study Koivisto et al. (2013) focused on the role of recurrent interactions for categorization of natural scenes and the PAS was used in an object substitution masking experiment. However, due to the small number of ‘no experience’ ratings, behavioral analyses were carried out by pooling together the ratings of the two lowest PAS categories (‘no experience’ and ‘brief glimpse’), showing how reduced perceptual awareness following masking affected categorization performance. Moreover, ERPs were investigated only for masked and unmasked trials, regardless of PAS rating. The situation is more complex when even a more fine-graded continuous scale is used to evaluate subjective visibility by moving a cursor on a horizontal bar where only the extremes are labeled (‘not seen’ and ‘maximal visibility’). Such a scale was employed in an attentional blink (Sergent et al., 2005) and in a masking (Del Cul et al., 2007) experiment; for both tasks the authors found that visibility ratings could be neatly divided into two categories, seen and not seen trials, without intermediate graded ratings, thus showing a sort of non-linear trend for visual awareness.

It thus seems clear that the difficult part is to get enough trials for each category of the scale, in order to perform analyses on all of them.

The aim of the present study is to explore the possible neural correlates of different grades of visual awareness. To do so, we studied the ERPs in response to reduced contrast visual stimuli at a detection threshold of about 50%. Participants had to judge the brightness of the stimuli and then qualitatively rate their visual experiences on the four-point PAS (Ramsøy and Overgaard, 2004). It was hypothesized that different grades of awareness may be reflected by different amplitudes of the components related to conscious perception (Koivisto and Revonsuo, 2003; Pins and Ffytche, 2003; Del Cul et al., 2007). More specifically, if consciousness is indeed a graded phenomenon, then a linear increase of the amplitudes of the components should be observed as a function of visual awareness. Furthermore, from the analysis of the intracranial generators we could draw some conclusions on where visual awareness emerges in the brain.

## Materials and Methods

### Participants

Twenty right-handed participants (13 females, mean age ± standard deviation: 22.5 ± 2.11) were recruited for the study. All reported normal or corrected-to-normal vision and no history of neurological or psychiatric disorders. They all gave their written informed consent to participate in the study. The study was approved by the local Ethics Committee and conducted in accordance with the 2013 Declaration of Helsinki. Data from six participants were excluded because there were not enough trials for analysis (<40 trials per condition) or because they showed an unequal distribution of the two stimulus types (lighter and darker) in one or more conditions. Two participants were not included in the study because of persistent noise in the EEG signal. The final sample was thus composed of 12 participants (11 females, mean age ± standard deviation: 23.08 ± 2.06).

### Stimuli

The stimuli were two-dimensional lighter or darker gray Gaussian patches with a standard deviation of 0.5°, presented for 34 ms on a gray background (8.01 cd/m^2^) at an eccentricity of 7° along the vertical meridian and of 12° along the horizontal meridian to the right of the fixation point. Two stimulus luminance values (one lighter and one darker than the background) were determined for each participant by means of a threshold assessment procedure: during this phase five different lighter luminance values (ranging from 6.65 cd/m^2^ to 7.60 cd/m^2^) and five different darker luminance values (ranging from 8.69 cd/m^2^ to 9.77 cd/m^2^) were used.

### Threshold Assessment

In a dimly lit testing room participants sat in front of a 17′ CRT monitor (resolution 1024 × 768, refresh rate of 85 Hz) placed at a viewing distance of 57 cm, with their head laying on a chin rest. The aim was to find two individual luminance values (one lighter and one darker) at which the participants reported to be aware of about 50% of the stimuli. The detection threshold was measured using the method of constant stimuli (Urban, 1910), where the preselected luminance values were presented in a randomized order in the periphery of the right visual field (see “Stimuli” for details). The procedure included 10 blocks: on each block, each luminance value was tested five times, resulting in a total number of 500 trials per participant. On each trial the stimulus appeared after a random interval (300–600 ms) following a brief 1000 Hz warning tone. The participants were asked to keep their eyes on a central fixation cross and press the spacebar whenever they saw a stimulus. At the end of the threshold assessment one lighter luminance value and one darker luminance value related to a 50%-detection threshold were chosen for each participant. These two luminance values were then used in the second phase of the experiment.

### EEG Experiment

**FIGURE 1:**
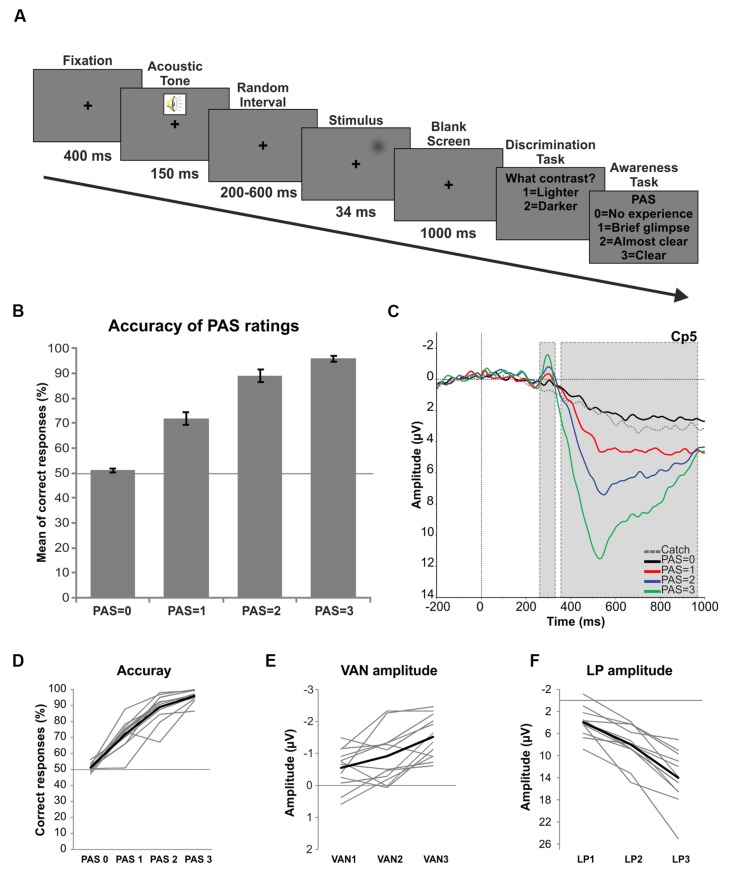
**Trial procedure and results.** **(A)** Experimental procedure: first, a fixation cross was presented for 400 ms followed by a warning acoustic tone lasting 150 ms. Then, a random interval ranging from 200 to 600 ms preceded the stimulus presentation (34 ms) in the periphery of the right visual field. After a 1000 ms pause participants had to discriminate the brightness of the stimulus (Discrimination task) and then rate the clarity of their perception on the PAS (Awareness task). **(B)** Behavioral results: mean percentage of correct responses for each level of the PAS. Error bars represent standard errors and the solid line (50%) chance level. **(C)** ERPs: grand average ERPs in response to each category of the PAS and catch trials for electrode Cp5. Gray dotted boxes indicate the components of interest (respectively VAN and LP). **(D)** Single subject behavioral data. The thick black line represents the mean of accuracy. **(E)** Single subject amplitudes of the VAN component as a function of the differences between each conscious condition (PAS = 1, 2, and 3) and the unconscious condition (PAS = 0). The thick black line represents the average of single subject amplitudes. **(F)** Single subject amplitudes of the LP component as a function of the differences between each conscious condition (PAS = 1, 2, and 3) and the unconscious condition (PAS = 0). The thick black line represents the average of single subject amplitudes.

Each trial started with a black fixation cross, followed 400 ms later by a 1000 Hz warning tone. After a random interval ranging from 200 to 600 ms a lighter or a darker gray Gaussian patch (whose luminance values were determined in the threshold assessment) was presented for 34 ms in the periphery of the right visual field. A 1000 ms pause was then followed by a response prompt asking the participants to judge the brightness of the stimulus as compared with the gray background, pressing a button for “lighter” and another button for “darker.” The participants were required to answer even if they did not see any stimulus. Then another response prompt asked the participants to rate the quality of their perception on the four-point Perceptual Awareness Scale (PAS; Ramsøy and Overgaard, 2004). The four PAS categories are: (0) no experience of the stimulus, (1) a brief glimpse, meaning that the participant saw something but could not discriminate the brightness of the stimulus, (2) an almost clear experience and (3) a clear experience. Responses were given by pressing four different buttons on the keyboard (**Figure 1A**). In order to verify that the participants used the PAS properly, at the end of the experiment we administered an open-ended questionnaire asking them to describe the criteria used for each category of the scale. The experimental session was divided into 20 blocks (66 trials each: 30 lighter, 30 darker and 6 stimulus-absent trials), thus yielding a total of 1320 trials. The order of the trials was fully randomized. Both the threshold assessment and the EEG experiment were programmed and run using E-prime (Psychology Software Tools, Inc., Pittsburgh, PA, USA^1^).

### EEG Recording and Event-Related Brain Potential (ERP) Analysis

EEG signal was continuously recorded with BrainAmp system (Brain Products GmbH, Munich, Germany – BrainVision Recorder) using a Fast’n Easy cap with 27 Ag/AgCl pellet pin electrodes (EasyCap GmbH, Herrsching, Germany) placed according to the 10–20 International System (O1, O2, P7, P3, Pz, P4, P8, Cp5, Cp1, Cp2, Cp6, T7, C3, Cz, C4, T8, Fc5, Fc1, Fc2, Fc6, F7, F3, Fz, F4, F8, Fp1, Fp2). Four additional electrodes were used for monitoring blinks and eye movements. Horizontal and vertical eye movements were detected respectively with electrodes placed at the left and right canthi and above and below the right eye. Other two extra electrodes served as ground (AFz) and online reference (right mastoid, RM). All scalp channels were then re-referenced oﬄine to the left mastoid (LM). Electrode impedances were kept below 5 kΩ. The digitization rate was 1000 Hz with a time constant of 10 s as low cut-off and a high cut-off of 250 Hz.

The continuous EEG signal was then processed off-line using Brain Vision Analyzer 2.0. Data were filtered with a high-frequency cutoff of 50 Hz (12 dB/octave) and a low-frequency cutoff of 0.1 Hz (12 dB/octave), and a 50 Hz notch filter was used to remove 50 Hz interference. Channels Fp1 and Fp2 were removed in all participants due to excessive noise. Independent component analysis (ICA) was applied to the whole dataset using the Infomax ICA algorithm (Bell and Sejnowski, 1995) in order to eliminate artefactual ICs. The EEG data were then cut into epochs of 1200 ms starting 200 ms before the onset of the stimulus and segmented trials were baseline corrected on the 200 ms pre-stimulus period. Before averaging, all segments were visually inspected and removed if contaminated by eye movements, blinks, strong muscle activity or excessive noisy EEG. The averaging was carried out for five different conditions: PAS = 0 (correct lighter and darker trials receiving a rating of 0 on the PAS), PAS = 1 (correct lighter and darker trials receiving a rating of 1 on the PAS), PAS = 2 (correct lighter and darker trials receiving a rating of 2 on the PAS), PAS = 3 (correct lighter and darker trials receiving a rating of 3 on the PAS) and Catch (stimulus-absent trials receiving a rating of 0 on the PAS). After pre-processing, the mean number of trials used for the average was 103 for PAS = 0, 75 for PAS = 1, 88.25 for PAS = 2, 65.92 for PAS = 3 and 57.83 for the Catch condition. Finally, for statistical analysis, data were downsampled to 250 Hz.

### Statistical Analysis

A repeated-measures analysis of variance (ANOVA) was carried out on the mean percentage of correct responses of each level of the PAS. A non-parametric binomial test was performed on the same measures to determine whether accuracies were significantly different from chance (50%).

Each conscious condition (PAS = 1, 2, and 3) was pairwise compared to the unconscious (PAS = 0) condition with the Mass Univariate ERP Toolbox (Groppe et al., 2011) implemented in Matlab by means of repeated measures, two-tailed *t*-tests on consecutive mean amplitude time windows of 20 ms, from 0 to 1000 ms at all electrode sites. For the three pairwise comparisons (PAS = 1 versus PAS = 0; PAS = 2 versus PAS = 0; PAS = 3 versus PAS = 0) the classic Benjamini and Hochberg (1995) false discovery rate (FDR) control procedure was applied with an FDR level of 5%.

Other two repeated-measures ANOVAs were then conducted in order to test whether the increment in amplitude of the VAN and the LP as a function of perceived clarity was linear. To do so, we evaluated by means of the trend analysis implemented as part of the analysis performed by the ANOVA in SPSS, whether a linear or non-linear (quadratic or cubic) function best represented data distribution by using polynomial coefficients. The first ANOVA was performed on the mean amplitude of the significant 20 ms time window (280–300 ms, VAN time range) of each level of the PAS for electrodes Cp5 and T7 separately (selected on the basis of the previous analysis). The second ANOVA was run on the mean amplitude of a significant 40 ms time window (510–550 ms, LP time range) of each level of the scale for electrode Pz only (selected according to the literature).

The generators contributing to the different levels of awareness as assessed on the PAS were defined using Scalp Current Density (SCD) maps, as implemented in BrainVision Analyzer 2.0. SCD maps are calculated from the Laplacian second derivative of the field potential that is directly proportional to the current density. This technique is independent from the reference electrode and mathematically eliminates the voltage gradients caused by tangential current flows, thus emphasizing the local contributions to the surface maps and providing a better visualization of approximate locations of intracranial generators. SCD topographic maps were computed from the spherical spline interpolation of the surface voltage recording (Perrin et al., 1989) for each conscious-unconscious difference. A fourth-order spherical spline was used with a spline-smoothing coefficient (*AAA*) of 1 × 10^-6^. In order to improve the signal-to-noise ratio and to account for inter-individual differences, SCD maps were created on the grand averages of the differences between each conscious condition (PAS = 1, PAS = 2, PAS = 3) and the unconscious condition (PAS = 0). As a result of the three pairwise comparisons, SCD analyses were performed on the VAN (280–300 ms) and LP (510–550 ms) time windows. The display gain of the maps was defined by visually inspecting the baseline period of the SCD maps (from -200 to 0 ms) to better appreciate the contribution of noise to the SCD topographies.

## Results and Discussion

### Behavioral Results

After the threshold assessment the mean luminance value chosen for lighter trials was of 9.23 cd/m^2^ and of 7.23 cd/m^2^ for darker trials. The mean percentage of catch trials receiving a rating of 0 on the PAS was 92.10% (*SD* = 5.19), thus revealing the reliability of the participants. For all trials, the mean percentage of PAS = 0 responses given by the participants was 42.66%, for PAS = 1 was 22.66%, for PAS = 2 was 22.88% and for PAS = 3 was 11.78%. A repeated-measures ANOVA conducted on the mean percentage of correct responses for each category of the PAS showed that, as visual awareness increased, also accuracy significantly increased [*F*(3,33) = 156.46, *p* < 0.01; linear trend *F*(1,11) = 1279.817, *p* < 0.01]. The mean percentage of correct responses for PAS = 0 was 51.15%, for PAS = 1 was 72.82%, for PAS = 2 was 85.68% and for PAS = 3 was 95.81% (**Figure 1B**). Interestingly, also at the single subject level this linear trend could be observed (**Figure 1D**), both for included and excluded (data not shown) participants. Finally, a non-parametric binomial test performed to determine whether the accuracy of each PAS level significantly differed from chance (50%) revealed that the performance when PAS = 1, PAS = 2 and PAS = 3 was significantly above chance level (all *p*s < 0.01), while for PAS = 0 it was not different from 50% (*p* > 0.05).

### ERP Results

Visual inspection of the grand average ERPs of each category of the PAS confirmed the presence of an early negative deflection (VAN) peaking at 300 ms at left channels followed by a later bilateral positive deflection (LP) starting at ∼400 ms (**Figure 1C**).

**FIGURE 2:**
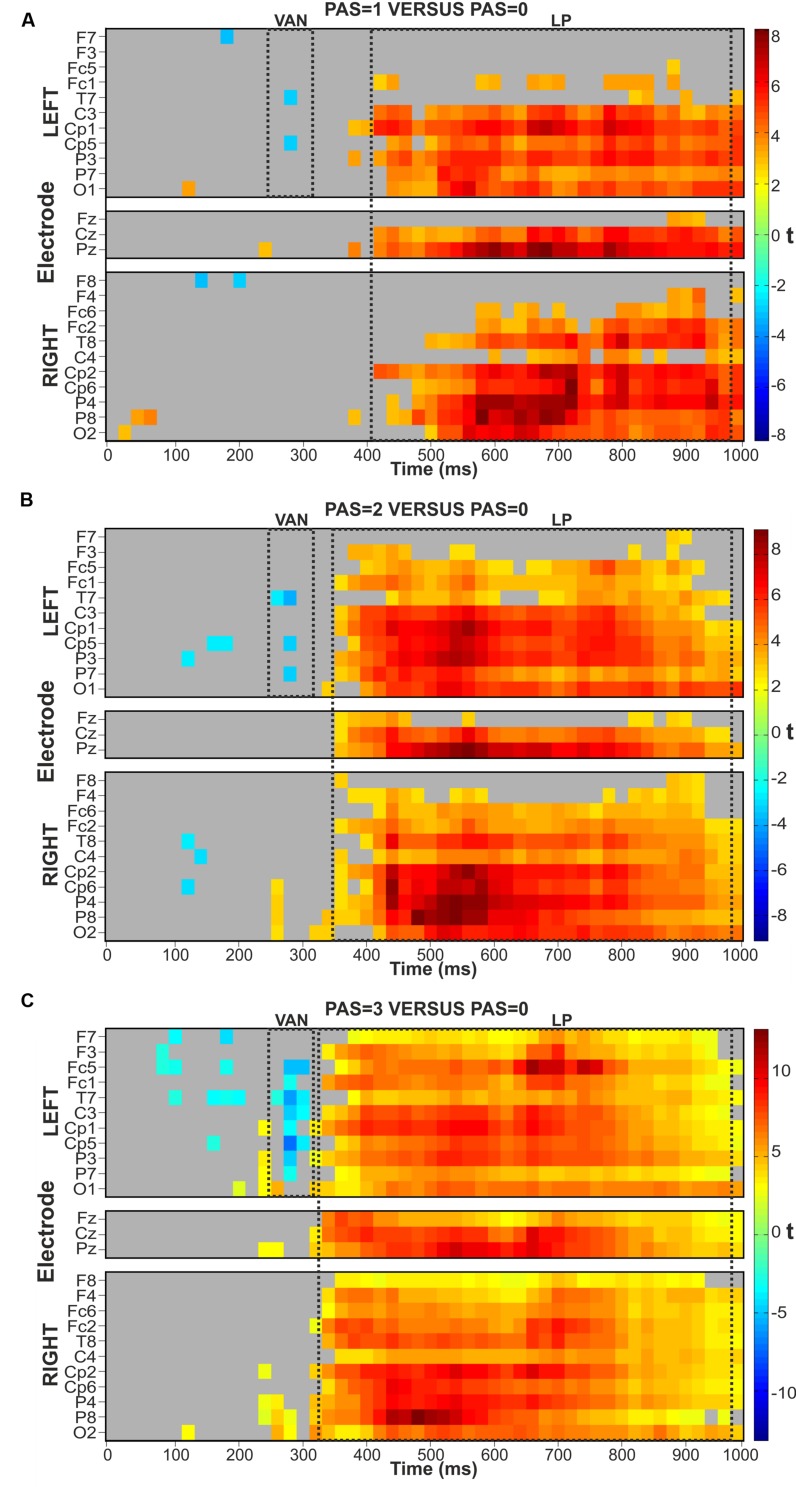
**Raster plots.** FDR-controlled *t*-test from Mass Univariate analyses of the three comparisons between each conscious (PAS = 1, 2, and 3) condition and the unconscious (PAS = 0) condition. *T*-tests were performed on the mean amplitude of consecutive time windows of 20 ms. **(A)** PAS = 1 versus PAS = 0. **(B)** PAS = 2 versus PAS = 0. **(C)** PAS = 3 versus PAS = 0.

To compare each conscious condition (PAS = 1, PAS = 2, PAS = 3) with the unconscious condition (PAS = 0) we analyzed the corresponding mean amplitudes by means of the Mass Univariate analysis (Groppe et al., 2011) in consecutive time windows of 20 ms, starting from 0 to 1000 ms after stimulus onset, at all electrodes. For the PAS = 1 versus PAS = 0 pairwise comparison, all significant FDR-corrected *p*-values were between 0.049056 and 0.001173 (**Figure 2A**), for PAS = 2 versus PAS = 0 between 0.049848 and 0.000320 (**Figure 2B**) and for PAS = 3 versus PAS = 0 between 0.047111 and 0.000040 (**Figure 2C**).

#### Visual Awareness Negativity

In the VAN time range (∼280–300 ms) the first conscious-unconscious pairwise comparison performed on PAS = 1 versus PAS = 0 conditions demonstrated that the ERP amplitudes differed significantly at electrodes T7 and Cp5 in the left hemisphere, contralateral to stimulus presentation (**Figure 2A**). The two-tailed *t*-tests of the second pairwise comparison on PAS = 2 versus PAS = 0 conditions revealed a significant difference again at left electrodes T7 and Cp5 but also at electrode P7 (**Figure 2B**). Finally, the two-tailed *t*-tests on PAS = 3 versus PAS = 0 conditions showed similar results to those obtained in the previous comparisons: the VAN effect was broader both in terms of time and number of significant electrodes (P7, P3, Cp5, Cp1, C3, T7, Fc1, and Fc5), spreading to left centro-parietal sites, as depicted in **Figure 2C**.

To test whether there was a linear increase in the amplitude of the VAN components, two repeated-measures analyses of variance were carried out for electrodes T7 and Cp5 on the mean amplitudes of each level of the PAS in the significant 20 ms time window (280–300 ms). We decided to choose these two channels since both of them resulted significant in all the three conscious-unconscious pairwise comparisons in the VAN time range. The two ANOVAs showed that for both electrodes the amplitude of the VAN increased as a function of visual awareness [T7: *F*(3,33) = 16.299, *p* < 0.01; Cp5: *F*(3,33) = 19.435, *p* < 0.01]. Interestingly, the analyses revealed a linear modulation in the increase of both electrode amplitudes [T7: linear trend *F*(1,11) = 34.858, *p* < 0.01; Cp5: linear trend *F*(1,11) = 54.263, *p* < 0.01] and such linear trend was evident in the data of each participant (**Figure 1E**).

Conscious conditions thus seemed to elicit more negative responses than the unconscious condition, as revealed by the presence of a reliable negative early component (VAN). The component peaked between 280 and 300 ms and was evident at left lateral posterior channels spreading to more parietal and central sites as visual awareness increased. Moreover, there was a linear graded modulation of the amplitude of the component as a function of the levels of visual awareness.

#### Late Positivity

Corresponding pairwise comparisons were performed in the LP time window. The two-tailed *t*-tests on PAS = 1 versus PAS = 0 conditions showed a widespread LP component starting at ∼420 ms post-stimulus and continuing until the end of the epoch (1000 ms), particularly over posterior and central sites, bilaterally (**Figure 2A**). The comparison performed on PAS = 2 versus PAS = 0 conditions revealed a widespread LP component that started at ∼360 ms and continued up to 1000 ms showing the most consistent effects bilaterally at posterior, central and partly also at prefrontal channels (**Figure 2B**). Finally, the two-tailed *t*-tests on the last conscious-unconscious comparison performed on PAS = 3 versus PAS = 0 found significant differences between the two conditions starting at ∼340 ms up to 1000 ms over all channels bilaterally (**Figure 2C**).

One repeated-measures ANOVA was performed for electrode Pz on the mean amplitudes of each level of the PAS in a 40 ms time window around the peak (510–550 ms) in order to test for a linear increase of the LP component. Given the widespread LP effect, channel Pz was chosen for analysis according to the literature (Del Cul et al., 2007). As for the VAN, the ANOVA revealed that the amplitude of the LP linearly increased with higher ratings of visual awareness [*F*(3,33) = 70.277, *p* < 0.01; linear trend *F*(1,11) = 103.177, *p* < 0.01] and this linear modulation was again found at the individual level, as shown in **Figure 1F**.

Responses to perceived trials were thus more positive than to unconscious trials. Such broad LP effect was bilaterally evident at central, posterior and lateral sites spreading also to prefrontal channels as visual awareness increased. Finally, the amplitude of the LP component was linearly modulated by visual awareness.

#### Scalp Current Density Maps

**FIGURE 3:**
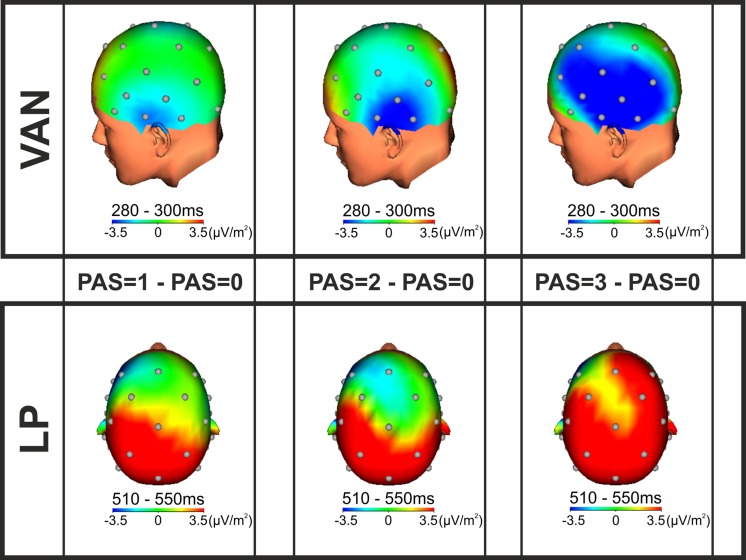
**SCD topographic maps.** SCD foci for the VAN (**upper**; time window from 280 to 300 ms) and LP (**lower**; time window from 510 to 550 ms) components performed on the grand average of the differences between each conscious condition (PAS = 1, 2, and 3) and the unconscious condition (PAS = 0).

Intracranial generators of the VAN and the LP were defined using SCD maps (**Figure 3**). According to the SCD topographies, at the lowest level of visual awareness (PAS = 1), the VAN component was consistent with left temporal generators, contralateral to stimulus presentation. The effect then spread to left posterior parietal areas at the intermediate level of awareness (PAS = 2) and finally activated a complex comprising also fronto-central generators at the highest level of conscious perception (PAS = 3). As regards the LP effect, the SCD topographies were consistent with bilateral posterior, lateral and central generators for the lowest and intermediate levels of visual awareness (PAS = 1 and PAS = 2), while for reports of clear experience (PAS = 3), current density foci were observed over different scalp areas including the prefrontal cortex and seemed larger in the right hemisphere. The intracranial generators of the phenomenal awareness (as assessed by the VAN) were thus found in the left temporal lobe, then the activation spread to posterior, central and prefrontal areas as a function of visual awareness. The LP component, interpreted to reflect access awareness, originated bilaterally in posterior, lateral and central areas extending to prefrontal regions as perceived clarity increased.

## General Discussion

In the present study, participants were required to rate the clarity of their perceptual experience of low-contrast stimuli on the four-point Perceptual Awareness Scale (Ramsøy and Overgaard, 2004). We found that their discrimination accuracy increased linearly as visual awareness increased. Moreover, ERP results revealed two electrophysiological components correlating with visual awareness. A negative early deflection, the VAN, peaking around 280–300 ms at lateral, parietal and central sites in the left hemisphere, followed by a later positive component, the LP, starting bilaterally 400 ms after stimulus onset over different scalp regions. As for accuracy, the amplitude of both components was found to increase linearly as a function of visual awareness as assessed on the PAS.

These results provide evidence that visual perceptual experience is characterized by a gradual increase of perceived clarity at both behavioral (accuracies) and neural (amplitudes) level. Such findings seem to be inconsistent with those by Del Cul et al. (2007), in which both accuracies and subjective ratings collected on a continuous scale where only the extremes were labeled exhibited a non-linear dichotomous distribution and the P300 was the only component whose amplitude varied with a similar sigmoidal trend to subjective ratings. The authors thus concluded that the P300 reflected the final stage of a process that led to an all-or-none reportability of a perceptual experience and they seem to allude to what Block (2005) defined “access consciousness.” However, it has been argued that the components in the P300 latency range and, thus, access consciousness might better reflect post-perceptual processes or consequences of consciousness, such as the confidence of the observer (Eimer and Mazza, 2005), different levels of accumulation of sensory evidence (Melloni et al., 2011) or working memory update (Polich, 2007). Given that the PAS is a measure of clarity of the perceptual experience and not a measure of confidence in response accuracy, our data would suggest that the LP might reflect the linear increase of the sensory evidence as the clarity of perceptual experience increases, in line with the interpretation given by Melloni et al. (2011).

Importantly, what proved to be the earliest most reliable correlate of phenomenal consciousness (Block, 2005), across different experimental paradigms and attentional manipulations, is the VAN, interpreted (Koivisto and Revonsuo, 2010) as the correlate of the actual content of perception as opposed to later post-perceptual processes (for a review see Railo et al., 2011). In agreement with this interpretation, the quality judgments given by our participants on the PAS were reflected in a concurrent linear modulation of the VAN amplitude, showing that different levels of cortical activity determined different levels of perceptual clarity. Similar results were found by Moutoussis and Zeki (2002) that showed how the difference between perceived and invisible stimuli depended on the strength of brain activation.

Our results are in line with findings obtained with different neuroimaging techniques. In an fMRI study (Christensen et al., 2006), reports of vague perceptual clarity versus clear experiences resulted in graded brain activity but also in unique patterns of cortical activation. As regards MEG, a recent experiment (Andersen et al., 2015) showed that occipital sources at the time window of the VAN could better decode graded levels of perceptual consciousness as assessed on the PAS. Taken together, all these pieces of evidence seem to support the graded nature of visual experience. Moreover, given its early latency, the VAN seems to be the component that better tackles the different degrees of perceived clarity of the phenomenal content.

An important point that deserves some considerations relates to the “where in the brain” perceptual awareness emerges. Our study, together with previous studies (e.g., Koivisto et al., 2013; Sandberg et al., 2013), seems to indicate that processes correlating with the graded contents of visual experience take place in temporal areas. The presence of early generators (VAN) in such posterior areas might be in line with data on phosphene perception (Bagattini et al., 2015): in this paper, the authors have proved how phosphene perception following occipital TMS stimulation is generated in the temporal cortex, while phosphene perception after parietal stimulation arises from the parietal regions. The fact that different generators have actually been found for the two different stimulation conditions thus confirms that the temporal and parietal cortices themselves are independent generators of conscious visual percepts. Both these and our results seem to be in favor of Zeki’s “micro-consciousness” proposal (Zeki and Ffytche, 1998), stating that local early activity in higher-order extrastriate regions plays a key role in generating visual perception. Indeed, it is evident from the analysis of intracranial sources that visual consciousness does not require later widespread fronto-parietal activation, as proposed by the Global Workspace Theory (GWT; Dehaene, 2014). This, again, is confirmed by phosphene studies (Bagattini et al., 2015), since a patient with a complete lesion of V1 showed differences for phosphene awareness only in an early time window, unlike healthy participants where differences were found also in a later phase in occipital and frontal areas. Likewise, recent MEG findings (Andersen et al., 2015) revealed that frontal sources at the P300 time range could not decode all PAS ratings. All these results seem to strengthen the assumption that such later frontal activity might support those consequences of consciousness (LP or access consciousness; Block, 2005) that are related to the components in the P300 time window (confidence, Eimer and Mazza, 2005; accumulation of sensory evidence, Melloni et al., 2011; update of working memory, Polich, 2007) and not perceptual awareness itself.

Another interesting aspect is that the PAS (Ramsøy and Overgaard, 2004) proved to be a good report measure to investigate different levels of perceptual clarity. Indeed, in the present study participants could use all the categories of the scale. Besides, we found that different levels of accuracy, and both the access properties (LP) and, more importantly, the actual phenomenal content of consciousness (VAN) differed depending on the levels of the PAS further corroborating the suggestion (Ramsøy and Overgaard, 2004) that each judgment given by the participants actually implies differences in processing. The implications related to such findings are important when considering blindsight patients. Blindsight follows a lesion in the primary visual cortex, resulting in a preserved ability to detect and discriminate visual stimuli presented in the blind field yet reporting no awareness of them: a phenomenon at first described as a case of unconscious vision (Weiskrantz, 1986). The exact mechanisms that are responsible for blindsight are still unknown but some patients with a huge lesion of V1 have been reported to exhibit some residual visual consciousness in their damaged hemifield (Barbur et al., 1993; Zeki and Ffytche, 1998). The use of a graded scale, such as the PAS, together with electrophysiological measures, might be helpful in discriminating patients showing a genuine blindsight phenomenon from those having residual conscious vision. In fact, using a dichotomous scale might not be sufficient to detect weaker forms of conscious perception, as already illustrated by Overgaard et al. (2008) in their seminal paper and more recently by Mazzi et al. (under review). In these studies, patient GR (Overgaard et al., 2008) and patient SL (Mazzi et al., under review), both suffering from a damage to the left occipital lobe, exhibited a blindsight behavior when tested with a binary seen/unseen scale, while when using the PAS, visual awareness was predictive of their performance, thus exhibiting conscious, yet degraded, vision. It could, thus, be predicted that patients diagnosed with degraded vision (as assessed on the PAS or another graded scale) would show similar components (VAN and LP) as the healthy participants in the present paper while genuine blindsight patients would not.

To summarize, we found that discrimination performance in a task with low-contrast stimuli increased as a function of visual awareness together with a linear amplitude modulation of the components correlating with the perceptual content (VAN) and post-perceptual processes (LP), suggesting that the nature of visual consciousness might be gradual. We also propose that the conscious phenomenal content of perceptual experiences emerges from the activation in temporal areas, as indicated by the topography of the intracortical generators of the VAN. Finally, the PAS seems to be an exhaustive measure in order to obtain more detailed subjective ratings.

## Author Contributions

CT, CM, and CB designed the experiment. CT conducted the experiment, analyzed data and wrote the manuscript. CM, CB, and SS supervised the analysis. SS conceived the study and revised the manuscript. All authors discussed the results and commented on the manuscript at all stages.

## Conflict of Interest Statement

The authors declare that the research was conducted in the absence of any commercial or financial relationships that could be construed as a potential conflict of interest.

## Footnotes

1 https://www.pstnet.com/eprime.cfm
